# The Flavonoid Apigenin Modulates Oligodendroglial Plasticity and Has a Neuroprotective Effect in Cerebellar Slice Cultures with Oxygen Glucose Deprivation

**DOI:** 10.3390/nu18071086

**Published:** 2026-03-28

**Authors:** Rodrigo Barreto Carreira, Cleonice Creusa dos Santos, Juciele Valeria Ribeiro de Oliveira, Nivia Nonato Silva, Victor Diogenes Amaral da Silva, Mauricio Moraes Victor, Arthur Morgan Butt, Silvia Lima Costa

**Affiliations:** 1 Laboratory of Neurochemistry and Cellular Biology, Institute of Health Sciences, Federal University of Bahia, Av. Reitor Miguel Calmon S/N, Salvador 40231-300, BA, Brazil; rodrigocarreira.rc@gmail.com (R.B.C.); cleonicemev@gmail.com (C.C.d.S.); juciele.valeria@ufba.br (J.V.R.d.O.); nivianonatoso@gmail.com (N.N.S.); vdsilva@ufba.br (V.D.A.d.S.); 2 Organic Chemistry Department, Chemistry Institute, Federal University of Bahia, Salvador 40170-115, BA, Brazil; mmvictor@ufba.br; 3 School of Medicine, Pharmacy and Biomedical Sciences, University of Portsmouth, Portsmouth PO1 2UP, UK; 4 National Institute of Translational Neuroscience (INNT), Federal University of Rio de Janeiro, Rio de Janeiro 21941-90, RJ, Brazil

**Keywords:** oxygen glucose deprivation, apigenin, oligodendrocytes, astrocytes, neuroprotection

## Abstract

**Background:** Apigenin, as a flavonoid, can be protective against oxidative damage in hypoxic events due to its antioxidant properties. Here, we have investigated the neuroprotective effects of apigenin in an ex vivo model of ischemic damage, using cerebellar slices from postnatal day (P)8–12 reporter mice to identify oligodendrocytes (SOX10-EGFP) and astrocytes (GFAP-EGFP). **Methods:** Apigenin (10 and 20 μM) was administered preventively at 60 min prior to and during inducing ischemic damage by oxygen and glucose deprivation (OGD); controls were maintained with glucose and normoxia (OGN). **Results:** OGD induced a marked retraction of oligodendroglial processes without reducing the oligodendrocyte number. This structural disruption was prevented by apigenin; notably, 10 μM apigenin blocked process retraction, whereas 20 μM did not, indicating a dose-dependent effect on the oligodendroglial morphology. Consistent with this, MBP and NF70 immunofluorescence analyses of axonal myelination demonstrated that OGD caused a significant loss of myelin sheaths, and this was prevented by pre-treatment with apigenin. In addition, apigenin prevented astrocyte reactivity induced by OGD, as assessed by increased GFAP-EGFP expression and decreased expression of glutamine synthetase. Moreover, immunofluorescence for calbindin indicated that apigenin protected Purkinje neurons from ischemic damage. **Conclusions:** These results demonstrate that apigenin is neuroprotective in ischemia and this is associated with modulation of astrocyte reactivity and maintenance of oligodendrocyte and myelin integrity.

## 1. Introduction

Stroke and associated brain ischemia [1] is a major cause of morbidity and mortality worldwide [2]. Ischemic attack due to cerebral infarction results in damage to brain tissue and neuronal cell death, with resulting neurological impairment [3,4], as observed in perinatal and adult stroke, as well as traumatic brain injury and multiple sclerosis [5,6].

In the initial phases of ischemic injury, astrocyte reactivity and microglial activation are induced by cytokines and chemokines, including interleukins (e.g., IL-1 and IL-6), kallikrein-related peptidase 6 and transforming growth factor beta (TGF-β) [7]. Astrocyte reactivity is generally characterized by an increased expression of glial fibrillary acidic protein (GFAP) and cellular hypertrophy, together with the formation of an astroglial barrier at the infarction site [8]. Activated microglia contribute to the inflammatory response and tissue repair through releasing pro- and anti-inflammatory cytokines, such as tumor necrosis factor alpha (TNF-α) and IL-1β [9].

Oligodendrocytes, the myelinating cells of the central nervous system [10,11], are highly susceptible to ischemic injury [12,13], which is a pivotal factor contributing to the deterioration of myelin and the degeneration of axons after ischemic episodes [14]. Reduced oxygen and glucose results in myelin damage and white matter pathology [15]. In the oxygen–glucose deprivation (OGD) model of ischemia, reduced viability and capacity for repair in oligodendrocytes and their precursors (OPCs) involves raised extracellular glutamate and over-activation of glutamate receptors [16,17].

Recent work from our group [18] demonstrated that the flavonoid agathisflavone (bis-apigenin) exerts robust neuroprotective effects in cerebellar slice models of neonatal ischemia, preventing oligodendroglial atrophy, myelin loss, astrocyte reactivity, and neuronal damage. Although structurally related, agathisflavone and apigenin differ markedly in their molecular complexity, bioavailability, and reported mechanisms of action. These distinctions justify the need to investigate apigenin independently, as its simpler monomeric structure and distinct pharmacological profile may confer complementary or more translationally accessible neuroprotective properties.

Apigenin (4′,5,7-trihydroxyflavone), a structural component of the biflavonoid agathisflavone, is a flavonoid found in several commonly consumed plant species such as cabbage, orange, tea, onion, and chamomile, and is widely recognized for its broad therapeutic potential [19,20]. Apigenin exhibits antioxidant, anti-inflammatory, and neuroprotective activities, making it a promising candidate for mitigating neurodegenerative processes [21]. Ischemic injury rapidly disrupts glial function—particularly in oligodendrocytes and astrocytes—which are central contributors to white matter vulnerability. Given that flavonoids can modulate oxidative and inflammatory pathways underlying these glial responses, apigenin stands out as a relevant compound to investigate in this context. This rationale supports our evaluation of whether apigenin can enhance glial resilience under OGD conditions.

In the present study, we examined the potential neuroprotective effects of apigenin in the ex vivo cerebellar slice OGD model of ischemia. The results show apigenin prevents ischemic damage to oligodendrocytes, myelin and neurons associated with altered astrocyte reactivity. These findings support evidence that apigenin is a promising natural compound for combatting neurodegeneration through its multifaceted neuroprotective properties.

## 2. Materials and Methods

### 2.1. Animals and Tissue

Mice of both sexes were used throughout, aged postnatal day (P)8–12, and a total of 16 animals were employed across all experimental procedures. Mice were of the C57BL/6 line and transgenic mice with Enhanced Green Fluorescent Protein (EGFP) reporter gene expression under the control of the astrocyte gene glial fibrillary acidic protein (GFAP) or the transcription factor encoded by the HMG-BOX 10 (SOX10) gene in oligodendrocytes and their precursors (gifts from Frank Kirchhoff, University of Saarland, Germany, and William Richardson, UCL, UK, respectively). Mice were housed in standard conditions within the University of Portsmouth animal facility and maintained on a 12 h light/dark cycle with controlled temperature and humidity, as well as ad libitum access to food and water. Litters were monitored daily by trained animal care staff, and no welfare concerns were identified prior to tissue collection. Mice were euthanized by exposure to carbon dioxide gas (CO_2_), followed by cervical dislocation and decapitation, according to the United Kingdom (Scientific Procedures) Act 1986 (ASPA) on the use of animals and approved by the University of Portsmouth Animal Welfare and Ethical Review Board (Process P93781054 approval on 24 March 2023). Brains were immediately dissected free in ice-cold artificial cerebrospinal fluid (aCSF) (consisting of 133 mM NaCl, 3 mM KCl, 2.24 mM CaCl_2_, 1.1 mM NaH_2_PO_4_, 1 mM MgCl_2_, 8.55 mM HEPES buffer, and 10 mM glucose, pH 7.3). Cerebellar slices 200 μm thick were made in the sagittal plane using a 5100 mz microtome vibratome (Camden Instruments LTD, Loughborough, UK), with settings of 80 Hz vibration frequency, 1.0 mm amplitude, and 0.15 mm/sec. speed, according to established protocols [22].

### 2.2. Flavonoid

Apigenin was provided by Professor Doctor Mauricio Victor (Research Group on Chemical Synthesis and Molecular Bioactivity, Chemistry Institute of the Department of Organic Chemistry, Federal University of Bahia (GPSQ/UFBA), Salvador, Brazil). Apigenin was obtained from the same batch that was synthesized and characterized in Victor et al. [23]. Full structural characterization of this batch (^1^H NMR, ^13^C NMR, IR) is reported in that publication. The same material was used in all experiments of the present study, it was produced from naringenin that was extracted from albedos of grapefruit peels, as detailed previously [23], and was stored at 4 °C as a 100 mM stock solution in dimethyl sulfoxide (DMSO, Sigma, St. Louis, MO, USA).

### 2.3. Cerebellar Brain Slices and OGD

**Figure 1:**
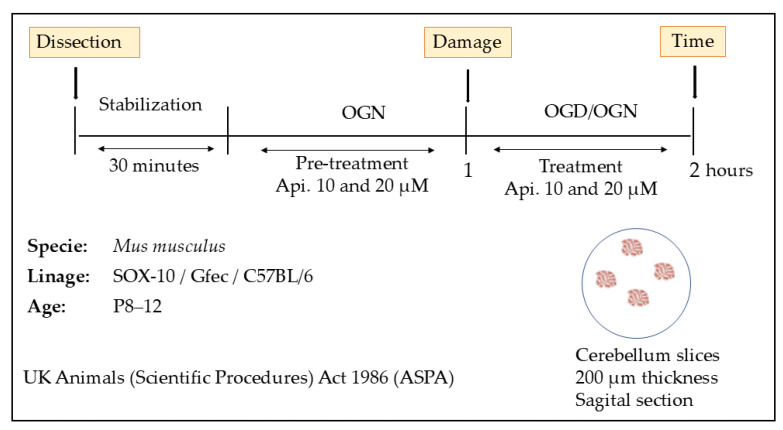
Representative scheme of experimental design for examining the effects of apigenin pretreatment on cerebellar slices exposed to acute oxygen and glucose deprivation. Api—apigenin, OGD—oxygen glucose deprivation, OGN—oxygen glucose normal.

Stock apigenin was dissolved in artificial cerebrospinal fluid (aCSF) at the time of the experiment, based on established methodology [24,25]. Cerebellar brain slices were stabilized in aCSF in 6-well plates under oxygen and glucose normal (OGN) conditions, then pre-treated with either aCSF + 0.01% DMSO sterile vehicle in controls (all experimental solutions contained DMSO vehicle) or apigenin (10 and 20 μM) under OGN conditions for 60 min in an incubator at 37 °C and 5% CO_2_. To maintain consistency with standard vehicle controls, all experimental and control solutions were adjusted to contain 0.01% DMSO, regardless of apigenin concentration; this concentration is widely used in ex vivo slice preparations and is considered non-toxic. Following pretreatment, slices were maintained for a further 60 min under continued OGN conditions or subjected to OGD, in which slices were maintained in aCSF without glucose (replaced with 10 mM sucrose) and transferred to a hypoxia chamber at 37 °C, 95% N_2_, and 5% CO_2_ (Figure 1). At the end of the experiment, slices were fixed in 4% paraformaldehyde for one hour and stored in PBS at 4 °C prior to immunofluorescence processing.

### 2.4. Immunofluorescence

Brain slices were permeabilized overnight at 4 °C in 1% PBS-T (1% Triton x-100 in PBS), prior to a blocking stage for 3 h using 20% bovine serum albumin (BSA) diluted in 0.01% PBS-T, then incubated in primary antibodies overnight at 4°, followed by incubation in appropriate secondary antibodies for 3 h at room temperature. All antibodies were dissolved in 1% normal goat serum (NGS) in PBS-T: anti-mouse myelin basic protein (MBP) (1:300, MAB 386, Burlington, MA, USA), anti-mouse neurofilament 70 (NF70) (1:300, MAB 1615, Burlington, MA, USA), mouse anti-calbindin D-28K (CB) (1:1000, Swant CB300PUR, Burgdorf, Switzerland) or rabbit anti-glutamine synthetase (GS) (Abcam ab49873, Darmstadt, Germany). Secondary antibodies: goat anti-rat (1:1000, Alexa Fluor 647) (Invitrogen A21247, Waltham, MA, USA), goat anti-mouse (1:1000, Alexa Fluor 488) (Invitrogen A11001, Waltham, MA, USA), goat anti-mouse (1:1000, Alexa Fluor 647) (Invitrogen A21235, Waltham, MA, USA), or goat anti-rabbit (1:1000, Alexa Fluor 647) (Invitrogen A21244, Waltham, MA, USA). Sections were counterstained with the nuclear dye Hoechst 33,342 at 5 μg/mL (Invitrogen, H1399, Waltham, MA, USA). Following washes in PBS, slices were mounted in VectaMount^®^ (H-5000-60, Newark, CA, USA).

### 2.5. Image Acquisition and Analysis

Confocal (Zeiss LSM 710) images were acquired using a ×20 objective and 10 μm z-stack intervals, keeping all parameters constant throughout; all slices were imaged and all images were included in the analysis. Across all experiments, each treatment group consisted of a minimum of 6 independent experimental units (FOVs), obtained from 2 to 3 non-overlapping fields per slice and 3 to 4 slices per condition. Relative fluorescence intensities were measured using Fiji-ImageJ version 2.14.0. Cell counts were performed in a constant field of view (FOV, 286.43 μm × 286.43 μm). A binary watershed filter was applied, and particles with an area between 20 and 200 μm^2^ were counted using Fiji-ImageJ version 2.14.0. The oligodendrocyte morphology was evaluated using the Analyze Skeleton Plugin (2D/3D) and cells were categorized as having few (<5), medium (5–9) or many (>9) processes per cells, as detailed previously [18].

### 2.6. Statistical Analysis

GraphPad Prism 10 was used for graph generation, while statistical analyses were performed in RStudio (version 2026.01.1+403). For each slice, 2–3 non-overlapping fields of view (FOVs) were acquired from anatomically distinct regions (e.g., different lobules or separate areas of the granular, molecular, or white matter layers). These FOVs were treated as within-slice subsamples. To avoid pseudo-replication, inferential statistics were conducted using hierarchical (mixed-effects) models in which treatment was specified as a fixed effect and slice identity as a random effect, with FOVs nested within slice; slice therefore represented the biological replicate. Model assumptions were assessed by inspection of residuals, and when necessary, outcomes were transformed or analyzed using non-parametric tests applied to slice-level summary values. Mixed-effects models were fitted using the restricted maximum likelihood (REML), and degrees of freedom for fixed effects were obtained using the Satterthwaite approximation implemented in the lmerTest package. Post hoc comparisons were adjusted for multiple testing using Tukey’s method. Statistical significance was defined as *p* < 0.05. Cell morphology data were compared using the chi-square test, with significance set at a 95% confidence level (*p* < 0.05). The number of slices analyzed per condition is reported for each experiment, and minor variation in the FOV number reflects the inclusion of only high-quality, non-overlapping anatomical regions.

## 3. Results

### 3.1. Apigenin Preserved Oligodendrocyte Integrity in OGD

**Figure 2:**
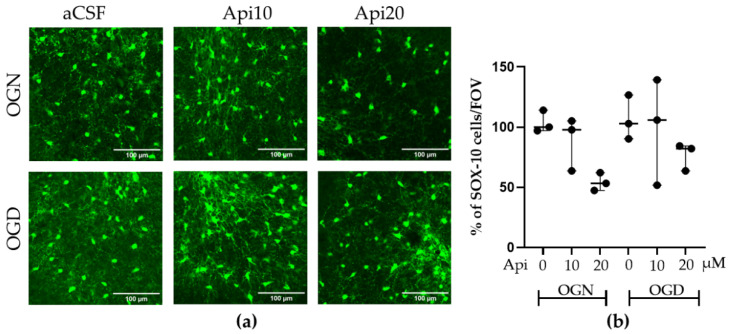
Effects of apigenin on oligodendrocyte numerical density following ischemic damage. (**a**) Representative confocal images of SOX10-EGFP+ oligodendrocytes in cerebellar slices treated with 10 or 20 μM apigenin in OGN or OGD conditions, as indicated; scale bar represents 100 μm. (**b**) Scatter plot graph of numerical density of SOX10-EGFP^+^ oligodendroglia in the cerebellar granular layer within a constant FOV. Data represents adjusted medians with 95% confidence intervals obtained from a linear mixed-effects model, with treatment as a fixed effect and slice identity as a random effect of 2 FOVs per slice, obtained from 3 cerebellar slices derived from 3 animals. The percentage values were normalized to the OGN-aCSF condition. The mixed-effects model did not detect a significant main effect of treatment (F(5,12) = 2.34, *p* = 0.106), and the Tukey-adjusted post hoc comparisons revealed no statistically significant differences between the groups.

The impact of OGD on oligodendrocyte integrity was assessed in postnatal mouse cerebellar slices ex vivo (Figure 2); SOX10-EGFP mice were used to identify the oligodendrocytes and OPC [26,27]. Cell counts of oligodendrocytes were performed in the granular layer because the vast majority (≥97%) of SOX10-EGFP+ cells are myelinating oligodendrocytes that are dispersed and can be individually identified (this is difficult in the white matter because oligodendrocytes are densely packed together). Oligodendrocyte numbers were unaffected by one-hour of OGD compared with OGN controls (Figure 2a,b); all experimental solutions contained a DMSO sterile vehicle that was used to dissolve apigenin. Additionally, cerebellar cultures exposed to 10 μM or 20 μM apigenin (Api10, Api20) showed no change in the number of oligodendrocytes, regardless of whether they were subjected to OGN or OGD (Figure 2a,b). Consistent with these observations, the linear mixed-effects model detected no significant main effect of treatment, and Tukey-adjusted post hoc comparisons revealed no statistically significant differences between the experimental groups.

**Figure 3:**
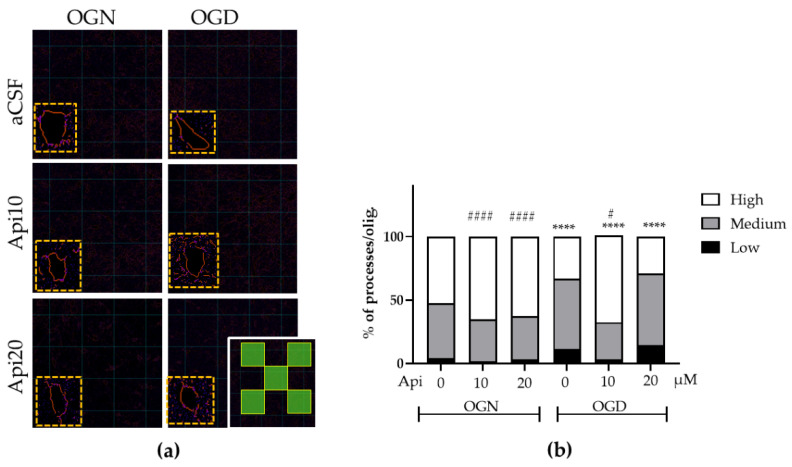
Effects of apigenin on oligodendrocyte morphology following ischemic damage. (**a**) Skeletonization of SOX10-EGFP+ oligodendrocyte in cerebellar slices treated with 10 or 20 μM apigenin in OGN or OGD conditions, as indicated; inset shows the quantified process area in SOX10-EGFP+ oligodendroglia, with a total counting area of 25,000 μm^2^ per image of cerebellar slices from SOX10-EGFP mice aged P8-12 were maintained in OGN or OGD and treated with apigenin at 10 or 20 μM. (**b**) Bar graph of the proportions of SOX10-EGFP+ oligodendroglia with low (0–4), medium (5–9), and high (>9) numbers of processes per cell. Data represents 2 FOVs per slice, obtained from 3 cerebellar slices derived from 3 animals, resulting in 6 images per group. FOVs correspond to spatially distinct microdomains. A global chi-square test performed on group-level totals indicated significant differences in the distribution of process complexity categories across experimental groups (χ^2^(10) = 64.02, *p* < 0.0001); a post hoc analysis was performed between OGN-aCSF and each experimental group, and between OGD-aCSF and each experimental group. The asterisks (*) and hashtags (#) indicate significance compared with OGN-aCSF and OGD-aCSF, respectively: # *p* ≤ 0.05; ****/#### *p* ≤ 0.0001.

Since ischemic damage had no effect on oligodendrocyte numerical density, we next examined oligodendrocyte morphology to determine whether ischemia caused process retraction [13]. EGFP fills the cell and enables visualization of oligodendrocyte processes (Figure 3). In control OGN-aCSF, oligodendroglia exhibited a complex-process-bearing morphology, with ~95% having ≥5 processes and over 50% having ≥10 processes, whilst cells with <5 processes were rarely observed (Figure 3b). Treatment with apigenin (10 or 20 μM) in OGN had no significant effect on oligodendrocyte morphological complexity, with ≥60% having a high number of processes (Figure 3b). In contrast, in slices subjected to one-hour of OGD-aCSF, oligodendrocytes displayed a significant decrease in morphological complexity compared with the OGN-aCSF controls (*p* < 0.001), revealing ischemia caused a marked retraction of oligodendroglial processes (Figure 3b). Apigenin (10 μM) blocked the detrimental effect of OGD conditions (*p* < 0.001 compared with OGN), with 99% of cells having ≥5 processes and 75% having ≥10 process (Figure 3b); this was also a significant improvement compared with OGN (*p* < 0.05). A concentration of 20 μM apigenin did not block the effects of OGD and the retraction of oligodendrocyte processes was significantly increased compared with OGN-aCSF (Figure 3b).

### 3.2. Apigenin Prevented Loss of Axonal Myelin Under OGD Conditions

**Figure 4:**
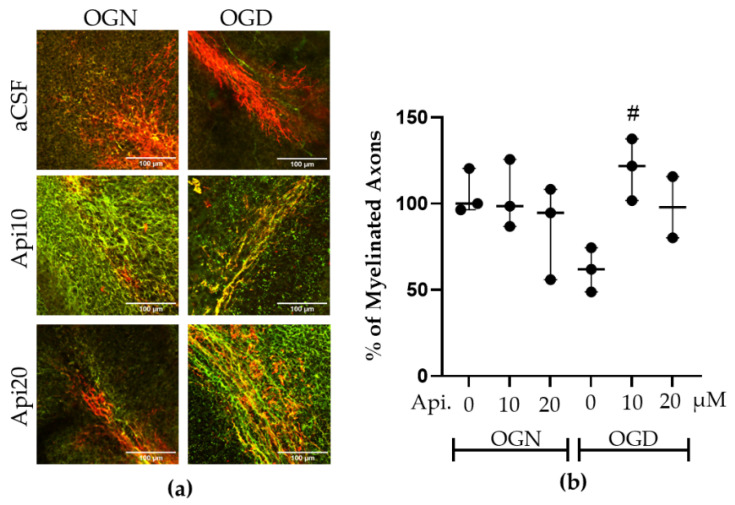
Effects of apigenin on cerebellar myelination following ischemic damage. (**a**) Representative confocal images of cerebellar slices immunolabelled for MBP (red) and NF70 (green) in the culture conditions indicated (colocalization appears yellow). (**b**) Scatter plot graph of the percentage of myelinated axons within a constant field of view (FOV) encompassing the myelinated white matter and granular layer, quantified as the Pearson correlation coefficient of colocalization between MBP and NF70 immunofluorescence. Data represents adjusted medians with 95% confidence intervals obtained from a linear mixed-effects model, with treatment as a fixed effect and slice identity as a random effect of 2–3 FOVs per slice, obtained from 3 cerebellar slices derived from 3 animals, resulting in a minimum of 6 images per condition. The number of FOVs varies slightly because only high-quality, non-overlapping anatomical regions were included; no images were excluded based on treatment or outcome. Pearson r values were Fisher-z-transformed prior to the statistical analysis and analyzed using a linear mixed-effects model with slice as a random factor (F(5, 9.24) = 3.53, *p* = 0.047). Post hoc Tukey comparisons were performed on Fisher-z values and back-transformed to Pearson r for presentation. The hashtag (#) indicates significance compared with OGN-aCSF: # *p* ≤ 0.05.

The retraction of oligodendrocyte processes caused by ischemia is an early indicator of the loss of oligodendroglial connections to their myelin sheaths [13]. Therefore, we next evaluated axonal myelination by quantifying the fluorescence intensity of double MBP-NF70 immunolabelling in a constant FOV encompassing the white matter and granular layers (Figure 4a). There was a decrease in axonal myelination by >40% in OGD compared with OGN controls, OGD-Api10 exhibited significantly higher MBP–NF70 colocalization than OGD-aCSF, indicating a partial restoration of axon–myelin association under ischemic conditions (Figure 4b); apigenin had no effect on myelination in OGN conditions.

### 3.3. Ischemia Causes a Loss of OPCs That Are Not Prevented by Apigenin Treatment

**Figure 5:**
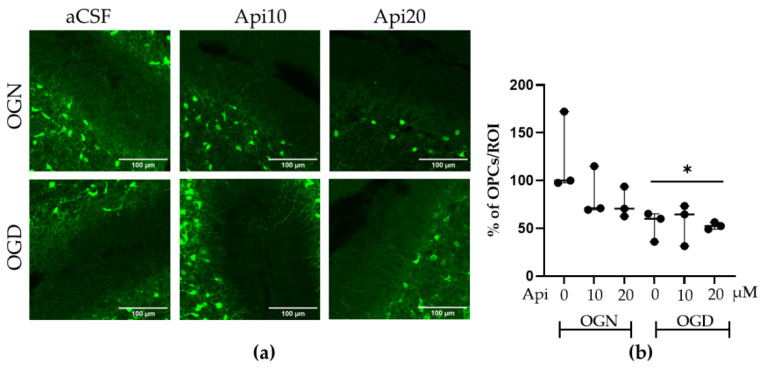
Effects of apigenin on OPCs following ischemic injury. (**a**) Representative confocal images of the unmyelinated cerebellar molecular layer showing SOX10-EGFP+ OPC in the different treatments groups, as labelled; the scale bar represents 100 μm. (**b**) Scatter plot graph of density of OPCs within a Region of Interest (ROI) in the molecular layer. Data represents adjusted medians with 95% confidence intervals obtained from a linear mixed-effects model, with treatment as a fixed effect and slice identity as a random effect of 2 FOVs per slice obtained from 3 slices per group, from 3 animals. The percentage values were normalized to the OGN-aCSF condition. The linear mixed-effects model with slice as a random factor detected a significant main effect of treatment (F(5, 12) = 3.85, *p* = 0.026). Post hoc Tukey comparisons revealed statistically significant differences between groups. The asterisks (*) indicates significance compared with OGN-aCSF: * *p* ≤ 0.05.

The cerebellar molecular layer is unmyelinated and all SOX10-EGFP+ cells are OPCs [28], and thus, we analyzed this region to determine the effects of OGD and apigenin on OPCs (Figure 5a); the opposite was the case for the white matter and granular layer used in the analyses of myelinating oligodendrocytes. Following one-hour of OGD, there was a significant (*p* ≤ 0.01) decrease in OPCs in the molecular layer, with a >50% loss of OPCs compared with OGN-aCSF controls (Figure 5b). Treatment with apigenin at 10 or 20 μM did not provide protection for OPCs, the amount of which was significantly fewer than the OGN-aCSF controls and did not statistically differ from OGD-aCSF (Figure 5b); OPCs were unaffected by apigenin in OGN.

### 3.4. Astrocyte Reactivity Is Induced by OGD and Prevented by Apigenin

**Figure 6:**
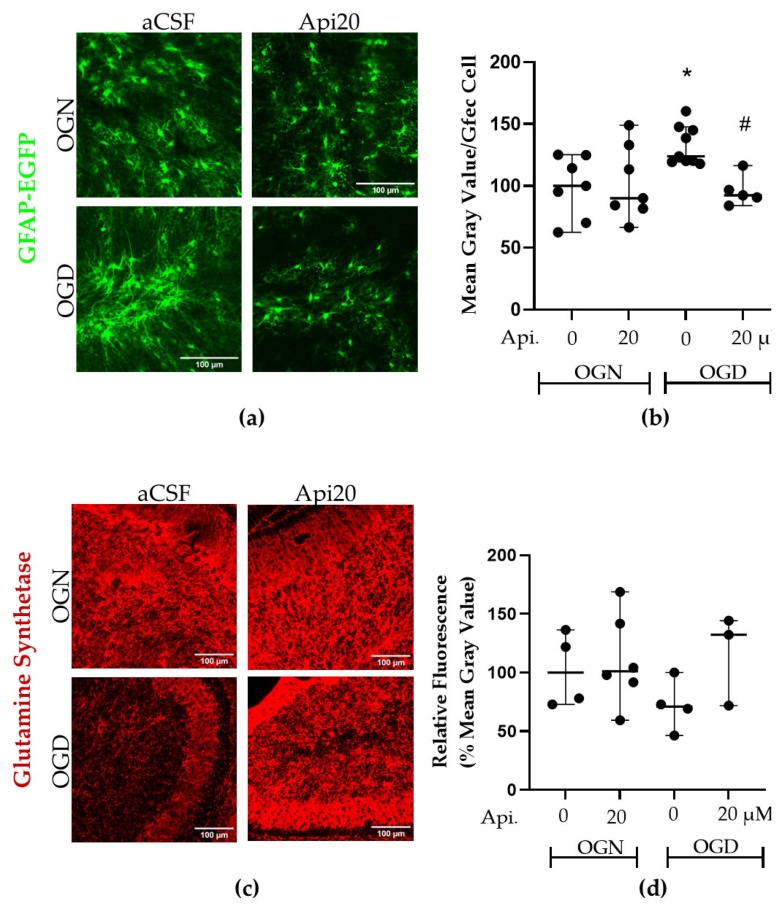
Effects of apigenin on astrocyte reactivity following ischemia. (**a**) Representative confocal images of cerebellar slices illustrating GFAP-EGFP expressing astrocytes (green) in the different treatment groups, as indicated; scale bars represent 100 μm. (**b**) Scatter plot of GFAP-EGFP fluorescence within a constant FOV in the granular layer. Data represent the median of 2–3 FOVs per slice, obtained from 5 to 9 cerebellar slices from 10 animals across 3 independent experiments. For this analysis, a slightly higher number of slices was included because images were derived from the GFEC, GFEC + glutamine synthetase, and GFEC + calbindin experimental datasets. No images were excluded based on treatment or outcome. Data are shown as medians with 95% confidence intervals. Percentage values were normalized to the OGN-aCSF condition. Statistical significance was tested using a one-way ANOVA performed on the mean FOV values per slice (F(3, 24) = 4.632, *p* = 0.0108) and post hoc Tukey’s multiple comparison test. The asterisk (*) and hashtag (#) indicate significance compared with OGN-aCSF and OGD-aCSF, respectively: */# *p* ≤ 0.05. (**c**) Representative confocal images of cerebellar slices immunofluorescence labelled for glutamine synthetase (GS) in the different treatment groups, as indicated; scale bars represent 100 μm. (**d**) Scatter plot graph of GS immunofluorescence within a constant FOV in the granular layer. Data represents adjusted medians with 95% confidence intervals obtained from a linear mixed-effects model, with treatment as a fixed effect and slice identity as a random effect of 2–4 FOVs per slice obtained from 3 to 6 slices per group from 3 animals. The percentage values were normalized to the OGN-aCSF condition. The linear mixed-effects model with slice as a random factor do not detect a significant main effect of treatment (F(3, 12.49) = 1.354 *p* = 0.3018).

Astrocytes respond to CNS trauma by a process termed reactivity, a common characteristic of which is the upregulation of GFAP, a widely recognized marker of astrocyte reactivity [29,30]. Glutamine synthetase (GS) is a key astrocyte enzyme involved in glutamate detoxification, which can be compromised in ischemia, contributing to glutamate-mediated excitotoxicity and neuronal death [31,32]. We therefore examined astrocyte responses to OGD using GFAP-EGFP reporter mice and immunofluorescence labelling for GS (Figure 6). Following one hour of OGD, there was a significant (*p* ≤ 0.05) increase in GFAP-EGFP expression compared with OGN-aCSF controls, and this was completely prevented by treatment with 20 μM apigenin; GFAP-EGFP expression in OGD-Api20 was significantly less than in OGD-aCSF (*p* ≤ 0.001) and not significantly different than control OGN-aCSF (Figure 6b); the increased GFAP-EGFP expression in OGD was also reduced by 10 μM apigenin (not illustrated), but this was not statistically significant (Figure 6b). Although the difference in GS expression between OGD and OGN-aCSF did not reach statistical significance, the Tukey post hoc test yielded *p* = 0.1113, indicating a trend toward reduced glutamine synthetase levels under acute oxygen-glucose deprivation, as shown in Figure 6d. Although reduced GS expression after OGD did not reach statistical significance (*p* = 0.1113), treatment with 20 μM apigenin appeared to prevent this decline, with levels comparable with controls. Apigenin had no effect on GFAP-EGFP or GS expression in OGN conditions. The results provide evidence that ischemia induces astrocyte reactivity, as defined by increased GFAP expression, and that this is prevented by apigenin treatment.

### 3.5. Apigenin Protects Purkinje Neurons Against OGD-Induced Injury

**Figure 7:**
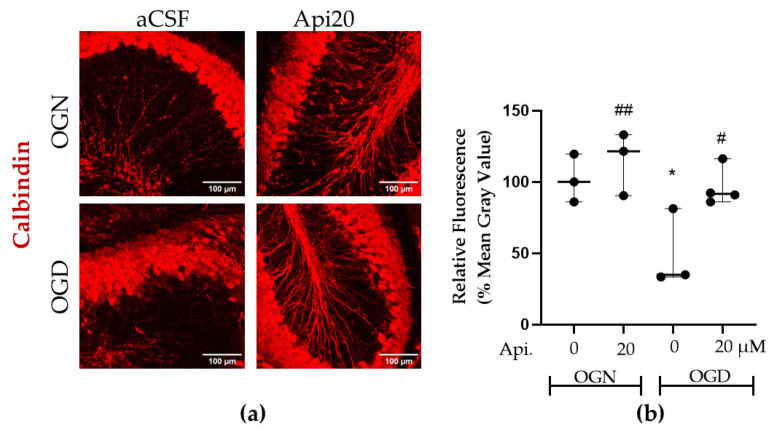
Effects of apigenin on Purkinje neurons in cerebellar slices following ischemia. Cerebellar slices from GFAP-EGFP mice aged P8-12 were maintained in OGN or OGD and treated with apigenin at 10 or 20 µM, and immunofluorescence labelled the Purkinje neuron marker calbindin D-28K. (**a**) Representative confocal images of cerebellar slices illustrating calbindin-immunopositive Purkinje neurons in the different treatment groups, as indicated; scale bars represent 100 μm. (**b**) Scatter plot graph of calbindin immunofluorescence within a constant FOV. Data represents adjusted medians with 95% confidence intervals obtained from a linear mixed-effects model, with treatment as a fixed effect and slice identity as a random effect of 2–4 FOVs per slice obtained from 3 to 4 slices per group from 3 animals. The percentage values were normalized to the OGN-aCSF condition. The linear mixed-effects model with slice as a random factor detected a significant main effect of treatment (F(3, 7.56) = 7.63, *p* = 0.011). Post hoc Tukey comparisons revealed statistically significant differences between groups. The asterisk (*) and hashtags (#) indicate significance compared with OGN-aCSF and OGD-aCSF, respectively: */# *p* ≤ 0.05, ## *p* ≤ 0.01.

The impact of OGD and apigenin on Purkinje neurons was examined because they are susceptible to ischemia [18,33], using immunolabelling for calbindin D-28K, which is a Ca^2+^-binding protein that plays a role in preventing neuronal death [34] and a recognized marker for cerebellar Purkinje neurons [35] (Figure 7). Compared with the OGN controls, ischemic injury with OGD significantly (*p* ≤ 0.01) reduced the expression of calbindin D-28K by 50%, and apigenin treatment (20 μM) completely prevented this OGD-mediated damage (*p* ≤ 0.05 compared with OGD-aCSF) (Figure 7a,b); apigenin had no effect on the OGN-aCSF controls.

## 4. Discussion

Apigenin is a structural component of the biflavonoid agathisflavone that has antioxidant properties [21,22]. In the present study, we provide evidence that apigenin is cytoprotective for neurons and glia ex vivo in cerebellar slices in the OGD model of ischemia. After 1 h, OGD induced a marked retraction of oligodendroglial cell processes and myelin loss without oligodendrocyte cell death during this acute period. In addition, there were astrocyte reactive changes in OGD, marked by increased GFAP expression and decreased glutamine synthetase, together with a loss of Purkinje neurons. Notably, treatment with apigenin, commencing 1 h prior to OGD and continued throughout 1 h of OGD, prevented these ischemia-induced degenerative changes and inhibited astrocyte reactivity, maintaining the expression of glutamine synthetase. These results demonstrate that apigenin is neuroprotective in the face of ischemic damage and this is associated with the modulation of astrocyte reactivity.

Oligodendrocyte disruption is evident in ischemic neuropathology [13,36,37]. The present study demonstrated that 1 h acute OGD ex vivo in cerebellar slices did not cause a loss of oligodendrocytes but induced early degenerative changes evidenced by retraction of their cellular processes. Ischemia has been shown to induce early onset process retraction in oligodendrocytes [18], and oligodendrocyte cell death does not occur until later, after 180 min of OGD [38]. It has been shown that OGD induced a decrease in SOX10-EGFP in the hippocampus [31], which is consistent with our findings of retraction of oligodendrocyte processes that connect to their myelin sheaths and are essential for maintaining myelin integrity. In support of this, we also observed a 40% decrease in axonal myelination, which was prevented by apigenin treatment. Although electron microscopy would be required to accurately determine changes in myelin *per se*, our results support studies showing that processes retraction and myelin disruption are the earliest overt oligodendroglial degenerative changes in ischemia and precede demyelination and potential cell death.

Ischemia-induced damage to oligodendrocyte processes and myelin has been shown to be at least partly mediated by glutamate acting on oligodendroglial NMDA receptors to cause Ca^2+^ influx [12,13]. Notably, oligodendrocytes exhibit an asymmetrical distribution of ionotropic glutamate receptors, with NMDA receptors concentrated in their processes and myelin sheaths, while AMPA and kainate glutamate receptors are mainly expressed on the cell body and their overactivation causes cell death [39]. There is evidence that apigenin inhibits the function of NMDA receptors, reaching 50% inhibition at a dose of 10 μM in rat cortical neurons [40]. Also, apigenin prevents the OGD-mediated loss of glutamine synthetase in reactive astrocytes, which is an important factor in glutamate-mediated excitotoxicity in ischemia [31,32]. The preservation of GS levels by apigenin, although not statistically significant, aligns with a potential role in enhancing astrocytic glutamate clearance, which may contribute—together with NMDA receptor inhibition—to its neuroprotective effects, and further by inhibition of the NMDA receptor, preventing ischemic damage to oligodendrocyte processes and myelin.

An interesting aspect of our findings is the clear concentration-dependent profile of apigenin. The protective effect was evident at 10 µM, whereas 20 µM failed to prevent the retraction of oligodendrocyte processes. This pattern aligns with the biphasic or hormetic responses frequently described for flavonoids, in which lower concentrations preferentially activate cytoprotective pathways, while higher concentrations may engage alternative signaling mechanisms or exert mild pro-oxidant actions [41,42]. While 10 μM apigenin prevented OGD-induced myelin loss, the effects of 20 μM on myelination remain to be determined. Conversely, only 20 μM protected Purkinje neurons, suggesting a differential dose–response profile across cell types. These observations suggest that the oligodendrocyte process complexity is particularly sensitive to subtle shifts in redox balance or intracellular signaling, reinforcing the importance of dose when defining the neuroprotective profile of apigenin.

In contrast to oligodendrocytes, OGD induced a loss of OPCs in the molecular layer of the cerebellum, and this was not prevented by apigenin; the molecular layer was analyzed because it does not contain myelinating oligodendrocytes, hence SOX10-EGFP+ cells are OPCs [28]. Our results indicate that cerebellar OPCs are more susceptible to ischemia than myelinating oligodendrocytes, in support of a previous study [18]. The loss of OPCs would be a major factor in limiting regeneration of myelinating oligodendrocytes and remyelination [36]. Notably, we have previously shown that agathisflavone, a dimer of apigenin, was able to protect OPCs from ischemic damage study [18], indicating it is more potent than apigenin and/or acts on alternative prosurvival pathways. The loss of OPC in OGD is consistent with their expression of AMPA receptors lacking the GluR2 subunit, which gives the receptors greater Ca^2+^ permeability [43], which would enhance the vulnerability of OPCs to glutamate-mediated excitotoxicity [43,44,45]. The lack of protection on OPCs by apigenin, compared with its robust preservation of mature oligodendrocytes, highlights that targeting additional mechanisms may be required to support OPCs, which is an aspect for future studies. Although apigenin effectively protected mature oligodendrocytes and preserved myelin integrity, its lack of effect on OPC survival suggests that these progenitors respond differently to acute ischemic stress. OPCs have a high metabolic demand and express distinct repertoires of glutamate and calcium-permeable receptors, which may render them more vulnerable to rapid energy failure and excitotoxicity than differentiated oligodendrocytes [46]. It is also possible that the short exposure window used here is insufficient to engage apigenin-responsive pathways in OPCs, or that these cells require additional trophic or anti-inflammatory support to withstand ischemic injury. Future studies will be important to clarify these possibilities, for example, by testing extended or post-injury treatment paradigms, evaluating combination approaches that target OPC-specific vulnerabilities, or examining whether apigenin influences later stages of OPC differentiation and remyelination.

Purkinje neurons are the primary neuronal population in the cerebellum [47], with their large cell bodies making up the Purkinje cell and their dendritic trees extending into the molecular layer where they form an extensively branched arborization [48]. The axons of Purkinje neurons make the myelinated axons we examined in the present study as they pass through the granular layer [48,49,50]. We found that 50% of cerebellar Purkinje neurons were lost after one-hour OGD and this was prevented by apigenin 20 μM. This supports previous studies indicating that Purkinje cell death in early postnatal stages is largely driven by glutamate excitotoxicity, primarily through Ca^2+^-permeable AMPA receptors in immature neurons [51,52], and the neuroprotective effects of apigenin are consistent maintaining astroglial glutamate regulation, rather than its actions on NMDA receptors, which are not expressed immature Purkinje neurons [53]. Damage to Purkinje neurons is also related to reduced GABA receptor function [54], and apigenin has been shown to promote the opening of GABA_A_ receptors [55], which would reduce Purkinje neuron excitotoxicity. In addition, progesterone has a protective effect on Purkinje cells [56] and—just as a phytoestrogen acts on estrogen receptors, which may be a further mechanism for the neuroprotective actions of apigenin, like that demonstrated for bis-apigenin (agathisflavone) [57]—this also prevented the death of Purkinje cells in OGD [18]. It should be noted that the prosurvival effects of apigenin on Purkinje neurons are likely to be extended to protection of their axons, which would be an important factor in the observed myelin protective effects described above.

Increased astrocyte expression of EGFP-GFAP in OGD is consistent with the classical pathological response of astrocytes, marked by changes in shape and gene expression [58]. However, it should be noted that astrocytes form a new glia limitans at the site of injury (often mistakenly called the glial scar), which limits inflammation spread in nervous tissue [59], and GFAP levels vary based on CNS location, injury proximity and the type of injury [60]. Apigenin prevented ischemic astrocyte reactivity by unresolved mechanisms, although it may involve pro-inflammatory mediators released by microglia that are activated in ischemia [61,62]. In ischemia, GFAP overexpression in astrocytes is regulated by several pathways, including JNK [63] and JAK-STAT3 [64], which may be mechanisms by which apigenin reduced GFAP expression. Apigenin may also act by inhibition of the Rho-associated protein kinase (RhoA)/ROCK pathway by hydrogen sulfide (H_2_S), which promotes the transition of astrocytes to a neuroprotective phenotype and modulates GFAP expression following ischemia/reperfusion injury [65]. Cysteine-rich angiogenic inducer 61 (CYR61) overexpression in astrocytes triggers nuclear factor kappa B (NF-κB) and the NLR family pyrin domain-containing 3 (NLRP3) inflammasome, leading to increased GFAP expression and neuroinflammation in hypoxic–ischemic brain damage [66]. The modulation of the astrocyte response is one of the mechanisms through which apigenin is likely to exert its neuroprotective effects following traumatic or inflammatory injury [25].

Altered expression of GS is characteristic of astrocyte reactivity [67,68], which is increased in acute cerebellar ischemia in post-mortem pediatric patients [69], as well as after three hours of ischemic injury in rats [70], but decreased in ischemia–reperfusion models [71,72]. The observed reduced expression of GS after one hour of ischemia and its prevention by apigenin is consistent with a previous study using bis-apigenin [18], which increased the expression of GS after excitotoxicity of glutamate in neuron–glia co-cultures [73]. In addition to astrocytes, GS is expressed by oligodendrocytes [74,75], which may have a bearing on the current findings.

A limitation of the present study relates to the structure of the acute slice preparation. Cerebellar slices obtained from each experiment were pooled and randomly allocated across treatment groups, which prevented retrospective identification of the animal of origin for each slice. Therefore, statistical analyses could not be performed at the animal level, and individual fields of view (FOVs) were treated as independent sampling units representing spatially distinct, non-overlapping anatomical microdomains within each slice. This approach is widely used in acute slice models, where pooling is necessary to ensure balanced allocation across conditions, but it carries an inherent risk of pseudoreplication. To mitigate this, all high-quality FOVs meeting predefined imaging criteria were included, resulting in a minimum of six images per group, and no images were excluded based on treatment or outcome. These considerations should be taken into account when interpreting the findings, although the consistency of the results across multiple cellular and molecular parameters analyzed supports the robustness of the observed neuroprotective effects of apigenin.

It is plausible that apigenin, as well as its dimer bis-apigenin, exerts protective effects against ischemic damage through multiple pathways and potential interactions in multiple cell types. Future research should elucidate which pathways are modulated by these flavonoids so that strategies can be directed to translate these findings into preventive therapies for groups at risk of ischemic damage. Bis-apigenin has demonstrated its myelinating properties and ability to induce remyelination in response to demyelinating damage, including in mouse cerebellar tissue [24,25]. Furthermore, its protective effects under ischemic conditions have been explored, showing that agathisflavone can preventively induce protection in acute cerebellar slices against OGD damage [18]. Together, these findings reinforce the relevance of flavonoid-based interventions as potential nutritional strategies to support glial and neuronal resilience under metabolic stress.

## 5. Conclusions

The results demonstrate that apigenin protects oligodendrocytes and neurons from ischemic damage. Additionally, we demonstrate that apigenin reduces ischemia-induced astrogliosis, notably maintaining the expression of GS—an essential enzyme for the detoxification of glutamate, which is critical in ischemic cytotoxicity. The results obtained indicate that apigenin has preventive therapeutic potential against ischemic lesions in neural tissue, which is relevant for contexts such as neonatal stroke, trauma and other neuropathological conditions.

## Data Availability

Data are contained within the article.

## Abbreviations

The following abbreviations are used in this manuscript:

aCSF	Artificial cerebrospinal fluid
ACTB	Actin beta
AMPA	α-amino-3-hydroxy-5-methyl-4-isoxazole-propionic acid
ANOVA	Analysis of variance
BDNF	Brain-derived neurotrophic factor
BSA	Bovine serum albumin
CB	Calbindin
CNS	Central nervous system
CYR61	Cysteine-rich angiogenic inducer 61
DMSO	Dimethyl sulfoxide
EGFP	Enhanced green fluorescent protein
FOV	Field of view
GAPDH	Glyceraldehyde-3-phosphate dehydrogenase
GFAP	Glial fibrillary acidic protein
GS	Glutamine synthetase
JAK	Janus kinase
JNK	c-Jun N-terminal kinases
MAPK	Mitogen-activated protein kinase
MBP	Myelin basic protein
NF70	Neurofilament 70
NF-κB	Nuclear factor kappa B
NG2	Chondroitin sulfate
NGS	Normal goat serum
NLRP3	NLR family pyrin domain containing 3
NMDA	N-methyl-D-aspartate
OGD	Oxygen glucose deprivation
OGN	Oxygen glucose normoxia
OPC	Oligodendrocyte precursor cell
PBS	Phosphate buffered saline
RhoA	Rho-associated protein kinase
ROI	Region of interest
STAT1	Signal transducer and activator of transcription 1
TGF-α	Transformation growth factor alpha
TNF-α	Tumor necrosis factor alpha
TRKB	Tyrosine receptor kinase B
